# BCL-2 Inhibitor Venetoclax Induces Autophagy-Associated Cell Death, Cell Cycle Arrest, and Apoptosis in Human Breast Cancer Cells

**DOI:** 10.2147/OTT.S281519

**Published:** 2020-12-31

**Authors:** Ali Alhoshani, Fahad O Alatawi, Fawaz E Al-Anazi, Ibraheem M Attafi, Asad Zeidan, Abdelali Agouni, Heba M El Gamal, Licia S Shamoon, Sarah Khalaf, Hesham M Korashy

**Affiliations:** 1 Department of Pharmacology & Toxicology, College of Pharmacy, King Saud University, Riyadh, Saudi Arabia; 2 Poison Control & Medical Forensic Chemistry Center, Jazan Health Affairs, Jazan, Saudi Arabia; 3 Department of Biomedical Sciences, College of Medicine, QU Health, Qatar University, Doha, Qatar; 4 Department of Pharmaceutical Sciences, College of Pharmacy, QU Health, Qatar University, Doha, Qatar

**Keywords:** venetoclax, MDA-MB-231 cells, breast cancer, apoptosis, BCL-2, cell cycle, autophagy

## Abstract

**Introduction:**

Venetoclax (VCX) is a selective BCL-2 inhibitor approved for the treatment of leukemia and lymphoma. However, the mechanisms of anti-cancer effect of VCX either as a monotherapy or in combination with other chemotherapeutic agents against breast cancer need investigation.

**Methods:**

Breast cancer cell lines with different molecular subtypes (MDA-MB-231, MCF-7, and SKBR-3) were treated with different concentrations of VCX for indicated time points. The expression of cell proliferative, apoptotic, and autophagy genes was determined by qRT-PCR and Western blot analyses. In addition, the percentage of MDA-MB-231 cells underwent apoptosis, expressed higher oxidative stress levels, and the changes in the cell cycle phases were determined by flow cytometry.

**Results:**

Treatment of human breast cancer cells with increasing concentrations of VCX caused a significant decrease in cells growth and proliferation. This effect was associated with a significant increase in the percentage of apoptotic MDA-MB-231 cells and in the expression of the apoptotic genes, caspase 3, caspase 7, and BAX, with inhibition of anti-apoptotic gene, BCL-2 levels. Induction of apoptosis by VCX treatment induced cell cycle arrest at G0/G1 phase with inhibition of cell proliferator genes, cyclin D1 and E2F1. Furthermore, VCX treatment increased the formation of reactive oxygen species and the expression level of autophagy markers, Beclin 1 and LC3-II. Importantly, these cellular changes by VCX increased the chemo-sensitivity of MDA-MB-231 cells to doxorubicin.

**Discussion:**

The present study explores the molecular mechanisms of VCX-mediated inhibitory effects on the growth and proliferation of TNBC MDA-MB-231 cells through the induction of apoptosis, cell cycle arrest, and autophagy. The study also explores the role of BCL-2 as a novel targeted therapy for breast cancer.

## Introduction

Breast cancer (BC) is one of the highest leading causes of cancer deaths.^1^,^2^ BC affects one in eight women and almost one million new cases of BC are identified every year worldwide.^3^ Thus, BC causes serious public health problems owing to its complexity, incidence, prevalence, mortality, heterogeneity, and, ultimately, the economic impact of therapy. The primary biomarkers for BC are the estrogen receptor (ER), progesterone receptor (PR), and human epidermal growth factor receptor 2 (HER2). The expression levels of these hormonal receptors allow for classification of BC into subtypes, with tumors lacking in ER, PR, and HER2 expression are classified as triple-negative breast cancer (TNBC).^4^ Unlike other cancer types, research has yet to confirm targeted therapy for TNBC, and the specific molecular underpinnings of the aggressiveness displayed by TNBC are not well understood. This consistently yields unfavorable medical prognoses and complicated treatment schemes.

Due to the overexpression of BCL-2 in BC, it was recognized as an effective prognostic marker for TNBC. Overexpression of BCL-2 occurs in approximately 41% of TNBC cases.^5^ Research has outlined a relationship between BCL-2 expression and increased chance of survival in TNBC patients,^6^ but the way in which BCL-2 could function as a therapeutic target for BC is not concrete. Several compounds responsible for targeting various components of the BCL-2 protein family are now being investigated in preclinical studies, along with clinical trials of TNBC. Among which, BH3 mimetic drugs hold the most significant level of potential in this respect.^7^ Notable studies have found that BH3 synthetic compounds display affinities for the pro-survival proteins. Among these BH3 mimetic compounds, venetoclax (VCX) received accelerated USA FDA approval for the treatment of acute lymphocytic leukemia in 2016 and chronic lymphocytic leukemia and lymphoma in 2019.^8^ VCX is a potent, selective, and orally available BH3 mimetic that serves as a BCL-2 inhibitor. VCX facilitates the restoration of the apoptotic capacity of cancer cells by displacement of BIM, a pro-apoptotic BCL-2 family protein, resulting in activation of apoptosis.^9^ With increased BCL-2 expression in BC and particularly TNBC, VCX may become a potential therapy for BC; however, the effects and the mechanisms mediating VCX anti-cancer activity need to be explored.

Although few previous studies have reported that VCX in combination with chemotherapeutic agents such as doxorubicin^10^ or dasatinib^11^ increased the apoptosis levels in different breast cancer cell lines, these studies lack the mechanisms involved particularly the role of autophagy and oxidative stress. Therefore, the current study aimed to test hypothesize that inhibition of BCL-2 signaling pathway by VCX induces BC apoptosis and autophagy-associated cell death. To test this hypothesis, we generally evaluated the potential effect of VCX on cell proliferation of three different BC cells with different molecular subtypes and investigated the molecular mechanisms of apoptosis, cell cycle, autophagy, and oxidative stress in TNBC MDA-MB-231 cells.

## Materials and Methods

### Chemicals

Venetoclax and doxorubicin were obtained from LC laboratories (Woburn, MA, USA). Bovine serum albumin and protease cocktail inhibitor reagents were purchased from Sigma Chemical Co. (St. Louis, MO, USA). High-Capacity cDNA Reverse Transcription kit and SYBR^®^ Green PCR Master Mix were obtained from Applied Biosystems^®^ (Foster City, CA, USA). Primary antibodies to target proteins and peroxidase-conjugated antibodies were purchased from Santa Cruz Biotechnology, Inc. (Santa Cruz, CA, USA). Chemiluminescence Western blot detection kits, Muse^®^ Count & Viability Kit, Muse^®^ Annexin V & Dead Cell Kit, Muse^®^ Caspase 3/7 Kit, Muse^®^ MitoPotential Kit, Muse^®^ Oxidative Stress Kit, and Muse^®^ Cell Cycle Kit were all obtained from EMD Millipore Co. (Billerica, MA, USA). All other chemicals were obtained from Fisher Scientific Co. (Toronto, ON, Canada).

### Cell Lines Culture and Treatment

Human breast cancer cell lines, MCF-7 (ER+), SKBR-3 (HER2+), and MDA-MB-231 (TNBC), were obtained from the American Type Culture Collection (Rockville, MD, USA). The cells were grown in Dulbecco’s Modified Eagle Medium with phenol red supplemented with Fetal Bovine Serum (FBS, 10%) and Antibiotic-Antimycotic (1%) using standard cell culture methods. Venetoclax and doxorubicin were prepared fresh in dimethyl sulfoxide (DMSO) before each experiment. The same concentration of DMSO was used as a control. Briefly, cells were seeded on 6- or 12-well culture plates to reach 80% confluency, after which they were treated for 24 h with DMSO or VCX (10, 25, and 50 μM), unless otherwise specified.

### Cell Viability and Proliferation Assay

BC cell proliferation was determined using Muse^®^ Cell Analyzer, EMD Millipore Co. (Billerica, MA, USA), according to manufacturer’s instruction, standardized to control probes as described previously.^12^ In brief, SKBR-3, MCF-7, and MDA-MB-231 cells were treated with a wide range of VCX concentrations (0.5, 1, 2.5, 5, 10, 25, 50, and 100 μM).^10^,^13^ After 24 h, the cells were collected by trypsinization and then incubated with Muse^®^ Count & Viability Kit, EMD Millipore Co. (Billerica, MA, USA). Both viable and non-viable cells were quantified, and the half-maximal inhibitory concentrations (IC_50_) were calculated using Sigma Stat^®^ Systat Software Inc. (San Jose, CA, USA).

### Mitochondrial Membrane Potential and Cellular Plasma Membrane Permeabilization Assay

The changes in mitochondrial potential and cellular plasma membrane permeabilization, early hallmarks of apoptosis and cell death, were determined with Muse^®^ Mitochondrial Kit (Millipore) as described previously.^14^ Briefly, MDA-MB-231 cells treated for 24 h with VCX were incubated with MitoPotential working solution for 20 min at 37°C in 5% of CO_2_ incubator. After incubation, 5 µL of Muse^®^ MitoPotential 7-ADD reagent was added and incubated for 5 min at room temperature. The percentage of four cell populations; live (mitopotential^+^/7-AAD^−^), depolarized live (mitopotential^−^/7-AAD^−^), dead (mitopotential^+^/7-AAD^+^), and depolarized dead (mitopotential^−^/7-AAD^+^) were measured using Muse^®^ Cell Analyzer, EMD Millipore Co. (Billerica, MA, USA), according to manufacturer’s instruction.

### Apoptosis Assay

The number and percentage of cells undergoing apoptosis were determined using Muse^®^ Annexin V & Dead Cell Kit, EMD Millipore Co. (Billerica, MA, USA) according to the manufacturer’s instructions and as described previously.^14^ Briefly, 80% confluent MDA-MB-231 cells were incubated for 24 h with VCX (10, 25, and 50 µM). The cells were collected by trypsinization, centrifuged at 300×g for 5 min, and then resuspended in 100 µL 1% FBS followed by incubation with Muse^®^ Annexin V & Dead Cell reagent in the dark. The percentage of healthy, apoptotic, and dead cells was counted with the Muse^®^ Cell Analyzer, EMD Millipore Co. (Billerica, MA, USA).

### Caspase 3/7 Activities

The quantitative measurements of apoptotic status based on caspase 3/7 activation were examined using Muse^®^ Caspase 3/7 Activation Kit, EMD Millipore Co. (Billerica, MA, USA), as described previously.^15^ Briefly, after 24 h treatment with VCX, cells were trypsinized, centrifuged at 300×g for 5 min, and then resuspended in 1X Assay Buffer, followed by incubation with 5 µL of caspase 3/7 reagent working solution for 30 min at 37°C in 5% of CO_2_ incubator. After incubation, 150 µL of Muse^®^ Caspase 7-ADD working solution was added and incubated at room temperature for 5 min in the dark. The percentage of caspase 3/7 positive cells was measured using Muse^®^ Cell Analyzer, EMD Millipore Co. (Billerica, MA, USA).

### Quantitative Real-Time Polymerase Chain Reaction (qRT-PCR)

After 24 h of treatment, total cellular RNA was isolated by a standard TRIzol method, Invitrogen Co. (Grand Island, NY, USA). The isolated RNA quantity and quality were maintained at 260/280 ratio of 1.8–2.0 using NanoDrop^®^ 8000 spectrophotometer, Thermo Fisher Scientific Inc. (Waltham, MA, USA). High-Capacity cDNA Reverse Transcription kit was used for cDNA synthesis in a Veriti^®^ Thermal cyclers (Applied Biosystems^®^).^16^ The mRNA expression levels of the target genes were quantified in QuantStudio^®^ 6 Flex Real-Time PCR System (Applied Biosystems^®^) using SYBR Green Universal Mastermix.^17^ Human primers for caspase 3, caspase 7, heme oxygenase-1 (HO-1), glutathione S Transferase A (GSTA), cyclin D1, E2F1, LC3, Beclin1, and β-actin (Table 1) were obtained from Integrated DNA Technologies (Coralville, IA, USA). The qRT-PCR data were analyzed using ∆∆CT method.^18^

**Table 1:** Primers Sequences Used for qRT-PCR Reactions

Gene	5ʹ→3ʹ Forward Primer	5ʹ→3ʹ Reverse Primer	Reference
*β-actin*	CCAGATCATGTTTGAGACCTTCAA	GTGGTACGACCAGAGGCATACA	[19]
*Caspase 3*	CGCAGACCTTGTGATATTCCAG	CGTTTCTTCCATCCTTCCAGG	[20]
*Caspase 7*	TGAGCCACGGAGAAGAGAAT	TTTGCTTACTCCACGGTTCC	[21]
*HO-1*	ATGGCCTCCCTGTACCACATC	TGTTGCGCTCAATCTCCTCCT	[19]
*GSTA*	TTGATGTTCCAGCAAGTGCC	CACCAGCTTCATCCCATCAAT	[20]
*Cyclin D1*	GATAGCCTTCGACCCAAGCA	ATGGCGGTGAGTGTCAGGAT	[20]
*E2F1*	ATGTTTTCCTGTGCCCTGAG	ATCTGTGGTGAGGGATGAGG	[22]
*LC3*	CATGAGCGAGTTGGTCAAGAT	TCGTCTTTCTCCTGCTCGTAG	[23]
*Beclin1*	TGAGGGATGGAAGGGTCTAAG	GCCTGGGCTGTGGTAAGTAATC	[23]

### Protein Extraction and Western Blot Analysis

Total protein isolated from MDA-MB-231 cells was quantified by Infrared (IR) spectroscopy Direct Detect^®^, EMD Millipore Co. (Billerica, MA, USA).^24^ Western blot analysis was performed as described before^24^ by separating approximately 30 μg protein in 10% sodium dodecyl sulfate-polyacrylamide gel electrophoresis (SDS-PAGE) gel and then transferred to a nitrocellulose membrane, Bio-Rad (Mississauga, ON, Canada). The protein blots were blocked overnight at 4°C, followed by incubation, at 4°C, with primary antibodies against target proteins followed by incubation with appropriate peroxidase-conjugated secondary antibody for 2 h at room temperature. The bands were visualized by chemiluminescence kits, EMD Millipore Co. (Billerica, MA, USA) using C-DiGit^®^ Blot Scanner, LI-COR Biosciences (Lincoln, NE, USA).

### Cell Cycle Distribution and Progression Analysis

Cell cycle distribution and progression were analyzed using Muse^®^ Cell Cycle Kit, EMD Millipore Co. (Billerica, MA, USA).^14^ Briefly, cells treated with VCX for 24 h were collected by trypsinization, centrifuged at 300×g for 5 min, and then resuspended in phosphate-buffered saline followed by incubation with 70% cold ethanol at −20°C for at least 30 min prior to staining with a premixed reagent propidium iodide (PI) and RNAse A. The DNA contents at all cell cycle stages were quantified using Muse^®^ Cell Analyzer, EMD Millipore Co. (Billerica, MA, USA).

### Reactive Oxygen Species (ROS) Production Assay

The cellular population undergoing oxidative stress was detected by measuring the levels of reactive oxygen species (ROS) using Muse^®^ Oxidative Stress Kit, EMD Millipore Co. (Billerica, MA, USA), as described previously^25^ and according to the manufacturer’s instructions. Briefly, pelleted cells treated with VCX were stained with Muse^®^ Oxidative Stress Working Solution that contains dihydroethidium (DHE), which is permeable and can react with superoxide anions undergoes oxidation to form the DNA-binding fluorophore ethidium bromide. The fluorescence produced was measured using Muse^®^ Cell Analyzer, EMD Millipore Co. (Billerica, MA, USA).

### Statistical Analysis

The statistical analysis of the results was performed using Sigma Stat^®^ for Windows, Systat Software Inc. (San Jose, CA, USA). Student’s *t*-test or one-way analysis of variance (ANOVA) followed by Student–Newman–Keul’s (SNK) tests were performed and the differences were considered significant when p <0.05.

## Results

### Effect of VCX Treatment on the Proliferation and Growth of Different Types of Breast Cancer Cell Lines

**Figure 1:**
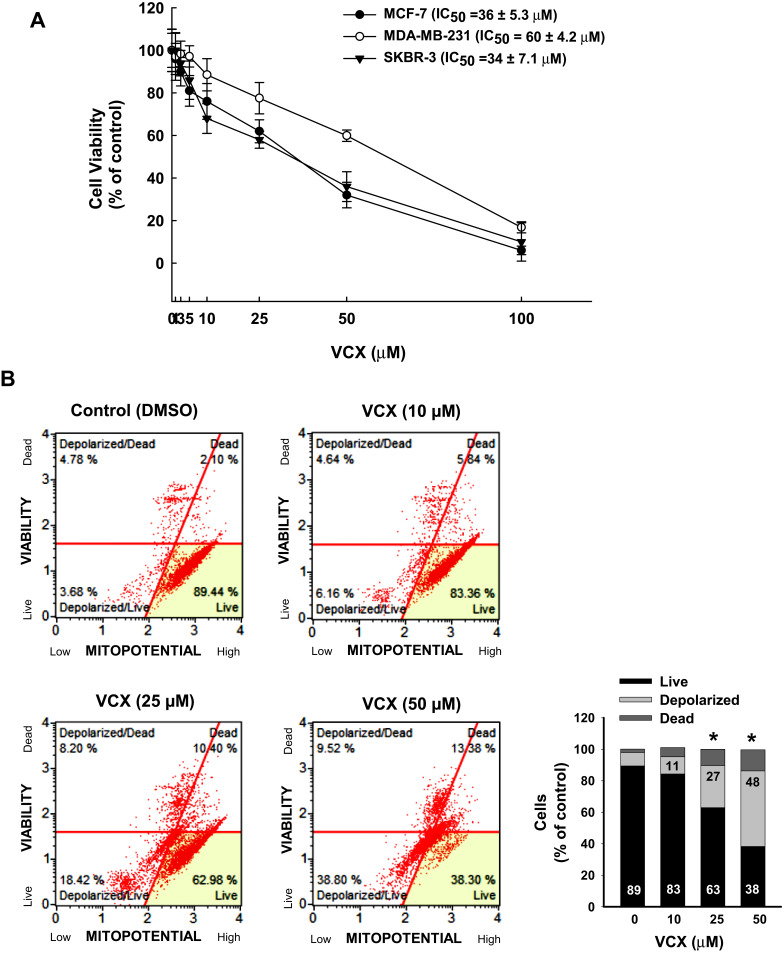
Effect of VCX on the proliferation and growth of human breast cancer cells. (**A**) Human breast cancer cell lines; SKBR-3, MCF-7, and MDA-MB-231 cells were treated for 24 h with a wide range of VCX concentrations. Cell proliferation and viability were determined by Muse^®^ Count & Viability assay. (**B**) MDA-MB-231 cells were treated with VCX (10, 25, and 50 μM) for 24 h. Thereafter, the percentages of live, depolarized, and dead cells were determined by Muse^®^ MitoPotential Kit using Muse^®^ Cell Analysis. The histogram represents the mean, (n = 3, triplicate). *P < 0.05 compared to control, VCX=0 μM, (ANOVA followed by SNK test).

We first assessed the ability of VCX to inhibit the growth and proliferation of three breast cancer cell lines of different molecular subtypes, SKBR-3 (HER2+), MCF-7 (ER+), and MDA-MB-231 (TNBC). The cells were incubated for 24 h with wide range of VCX concentrations (0.5, 1, 2.5, 5, 10, 25, 50, and 100 μM), those concretions were selected based on previous studies,^10^,^13^ after that cell proliferation was determined as described in the Materials and Methods section. Figure 1A shows that VCX at concentrations up to 5 μM did not significantly alter cell viability and proliferation of all tested cells. However, a significant decrease in cell proliferation was observed at VCX concentrations 10, 25, 50, and 100 μM in all tested cells in a concentration-dependent manner (Figure 1A). The IC_50_ of VCX for MDA-MB-231 was approximately 60 ± 4.2 μM, whereas MCF-7 and SKBR-3 cells were more sensitive to VCX inhibitory effects with IC_50_ of 36 ± 5.3 and 34 ± 7.1 μM, respectively (Figure 1A). Based on cell viability/proliferation studies, the concentrations 10, 25, and 50 μM have been selected to be utilized in all subsequent studies using TNBC MDA-MB-231 cell line as a study model.

### Effect of VCX Treatment on Cell Mitochondrial Potential and Cellular Plasma Membrane Permeabilization

Depolarization of the mitochondrial membrane potential prevents calcium entry into the mitochondria causing cell viability reduction, and this is considered as an indicator for early apoptosis. To test whether the inhibitory effect of VCX on MDA-MB-231 cell proliferation is due to the depolarization of the mitochondria membrane, we examined the effect of VCX on the mitochondrial potential and cellular plasma membrane permeabilization, a marker for cell death.^26^ For this purpose, MDA-MB-231 cells were treated for 24 h with VCX (10, 25, and 50 μM), and then the percentage of live cells with intact mitochondria, depolarized live and dead cells, and dead cells with intact mitochondria were determined by flow cytometry. Figure 1B shows that VCX treatment at 10 μM concentration did not significantly alter the mitopotential and membrane permeability. However, higher VCX concentrations (25 and 50 μM) significantly increased the percentage of depolarized (live and dead) cells up to 27% and 48%, respectively, as compared to the control (8%). In addition, the percentage of live cells was significantly decreased to 38% at the highest concertation tested (50 μM) (Figure 1B).

### Effect of VCX Treatment on Apoptosis

**Figure 2:**
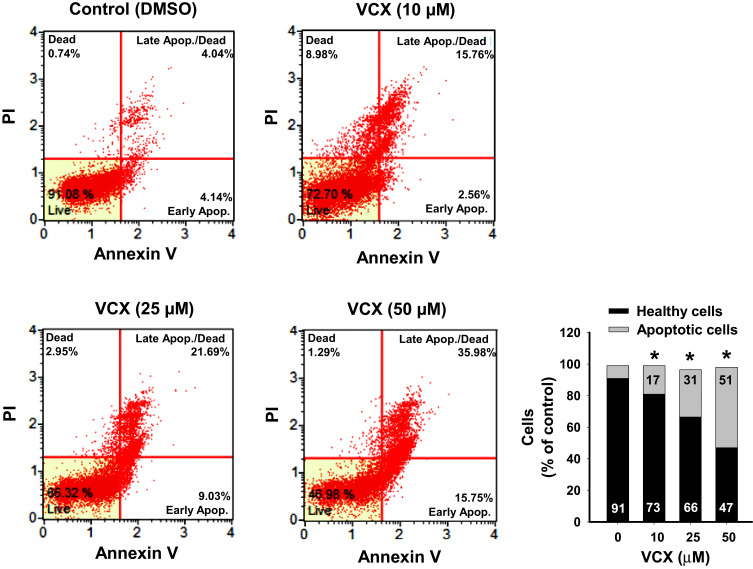
Effect of VCX treatment on the apoptosis level in MDA-MB-231cells. MDA-MB-231 cells were treated for 24 h with VCX (10, 25, and 50 μM). Thereafter, the percentage of cell undergoing apoptosis were determined using Muse^®^ Annexin V & Dead Cell Kit. The histogram represents the mean, (n = 3, triplicate). *P < 0.05 compared to control, VCX=0 μM, (ANOVA followed by SNK test).

To explore the mechanisms of the VCX inhibitory effect on MDA-MB-231 cell growth and proliferation, we investigated whether this effect could be attributed to increased apoptotic and/or necrotic cell population. Treatment of MDA-MB-231 cells for 24 h with VCX (10, 25, and 50 μM) significantly increased the percentages of apoptotic cells (early and late) at all tested concentrations, in a concentration-dependent manner, by approximately 2-, 4-, and 6-fold, respectively, as compared to the control (Figure 2).

**Figure 3:**
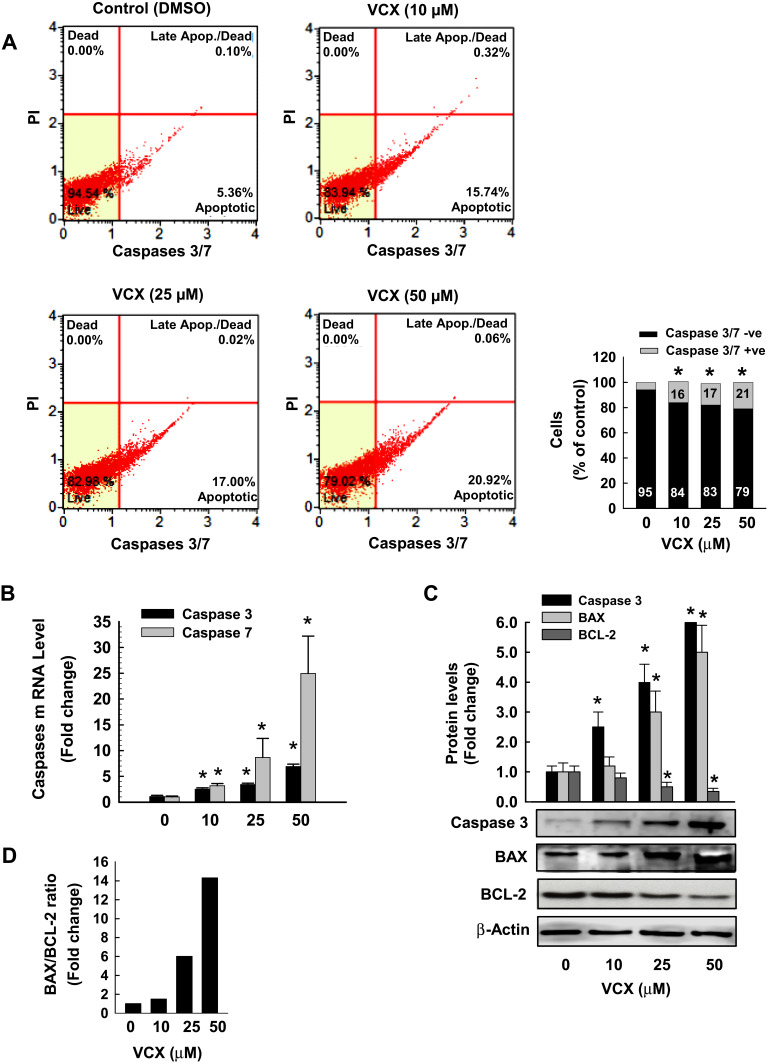
Effect of VCX treatment on the activity and expression of pro- and anit-apoptotic markers in MDA-MB-231cells. MDA-MB-231 cells were treated for 24 h with VCX (10, 25, and 50 μM). (**A**) The percentage of cells expressing caspases 3/7 was determined using Muse^®^ Caspases 3/7 Kit. The histogram represented the mean, (n = 3, triplicate). *P < 0.05 compared to control, VCX=0 μM, (ANOVA followed by SNK test). (**B**) Caspases 3 and 7 mRNA levels were quantified using qRT-PCR and normalized to β-actin housekeeping gene. Triplicate reactions were performed for each experiment. The values represented the mean of fold change ± SEM, (n = 6). *P < 0.05 compared to control, VCX=0 μM, (ANOVA followed by SNK tst). (**C**) caspase 3, BAX and BCL-2 protein levels were determined by Western blot analysis. One of three representative experiments is shown. The values represented the mean of fold change ± SEM, (n = 3). *P < 0.05 compared to control, VCX= 0 μM, (ANOVA followed by SNK test). (**D**) Histogram represents BAX/BCL-2 ratio.

To further examine whether increased the percentage of apoptotic cells by VCX treatment is associated with changes in the activity and expression of pro-apoptotic and anti-apoptotic markers, we measured the effect of 24 h treatment of VCX on the expression of caspases 3/7, BAX, and BCL-2 in MDA-MB-231 cells. Our results show that VCX 25 and 50 μM treatment significantly increased caspases 3/7 activity using flow cytometry (Figure 3A) and their mRNA (Figure 3B) levels in a concentration-dependent manner. At the protein level, VCX treatment induced the pro-apoptotic caspase 3 and BAX proteins by approximately 6- and 5-fold, respectively, whereas dramatically inhibited the anti-apoptotic BCL-2 protein by more than 65% at the highest concentrations tested, 50 μM (Figure 3C). The BAX:BCL-2 ratio was increased by VCX in a concentration-dependent manner reached up to 7- and 14-folds at concentrations 25 and 50 μM, respectively (Figure 3D).

### Effect of VCX Treatment on Cell Cycle Phases and Genes

**Figure 4:**
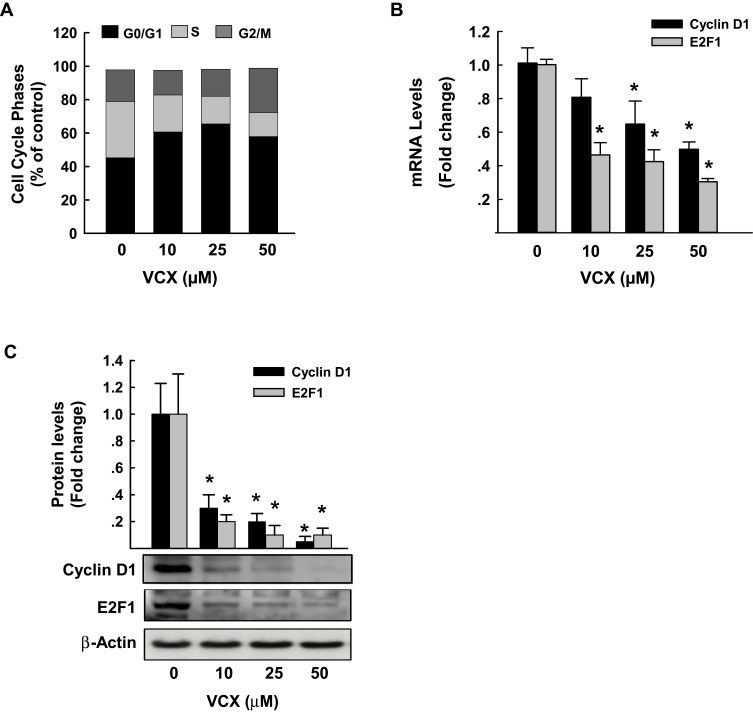
Effect of VCX treatment on MDA-MB-231 cell cycle phases and genes. MDA-MB-231 cells were treated for 24 h with various concentration of VCX (10, 25, and 50 μM). (**A**) Cell cycle phases were determined by Muse^®^ Cell Cycle assay. Values are presented as the mean of percentage of live cells (n = 3, triplicate). (**B**) Cyclin D1 and E2F1 mRNA levels were quantified using qRT-PCR and normalized to β-actin housekeeping gene. Triplicate reactions were performed for each experiment. The values represented the mean of fold change ± SEM, (n = 6). *P < 0.05 compared to control, VCX=0 μM, (ANOVA followed by SNK test). (**C**) Cyclin D1 and E2F1 protein expression levels were determined by Western blot analysis. One of three representative experiments is shown. The values represented the mean of fold change ± SEM, (n = 3). *P < 0.05 compared to control, VCX=0 μM, (ANOVA followed by SNK test).

To examine whether the arrest of the cell cycle is contributing to the inhibitory effect of VCX on MDA-MB-231 cell proliferation and growth, we performed a cell cycle analysis by flow cytometry. Figure 4A shows that VCX treatment caused changes in cell cycle progression at all tested concentrations. For example, VCX 50 μM significantly increased the percentage of cell population in the G0/G1 phase to 65% (50% increase) as compared to control (43%), whereas decreased cell population in the S phase to 16% (50% decrease) as compared to control (34%). No significant changes in G2/M phase were observed in response to VCX treatment (Figure 4A).

To further determine whether the cell cycle arrest by VCX is attributed to a downregulation in the expression of cell cycle proliferative genes, we quantified the mRNA and protein expression levels of cyclin D1 and E2F1, well-known cell cycle genes, by qRT-PCR and Western blot analyses, respectively. Data in Figure 4B illustrate that VCX significantly inhibited cyclin D1 and E2F1 mRNA expression by approximately 55% and 70%, respectively, at VCX 50 μM concentration. At the protein expression level, cyclin D1 and E2F1 proteins were markedly inhibited by all tested VCX concentrations, reached up to 90% by VCX 50 μM as compared to control (Figure 4C).

### Effects of VCX Treatment on Oxidative Stress

**Figure 5:**
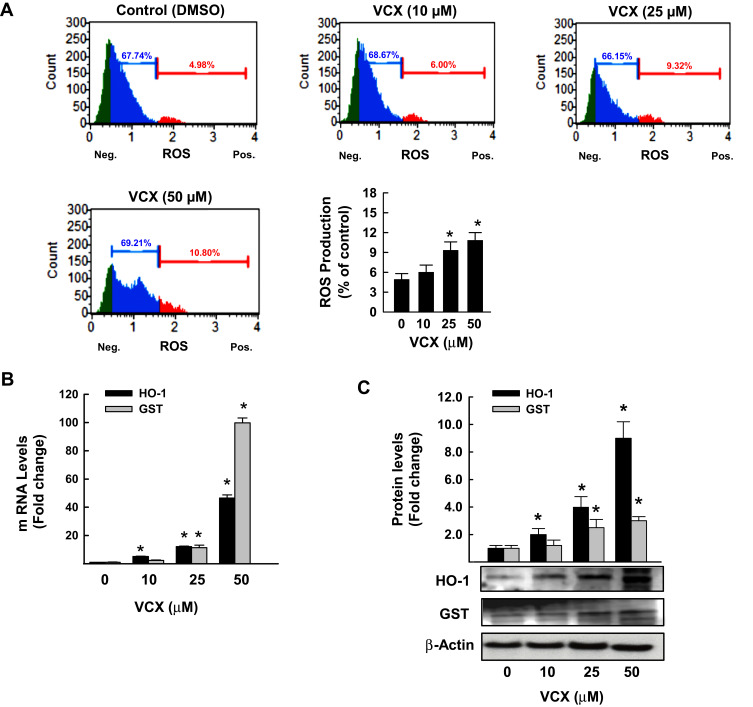
Effect of VCX treatment on oxidative stress in MDA-MB-231cells. MDA-MB-231 cells were treated for 24 h with various concentrations of VCX (10, 25, and 50 μM). (**A**) Production of ROS was determined by Muse^®^ Oxidative Stress Kit. The values represented the mean ± SEM, (n = 3, triplicate). *P< 0.05 compared to control, VCX=0 μM, (ANOVA followed by SNK test). (**B**) HO-1 and GSTA mRNA levels were quantified using qRT-PCR and normalized to β-actin housekeeping gene. The values are presented as mean ± SEM, (n = 6, triplicate). *P < 0.05 compared to control, VCX=0 μM, (ANOVA followed by SNK test). (**C**) HO-1 and GSTA protein expression levels were determined by Western blot analysis. One of three representative experiments is shown. The values represented the mean ± SEM, (n = 3). *p< 0.05 compared to control, VCX=0 μM, (ANOVA followed by SNK test).

We tested the role of oxidative stress in VCX inhibitory effect on cell proliferation by first measuring the percentage of MDA-MB-231 cells generating ROS and second by quantifying the mRNA and protein expression levels of oxidative stress-mediated genes, HO-1 and GSTA. The flow cytometry analysis data in Figure 5A show that VCX 25 and 50 μM treatment significantly increased the percentage of cells generating ROS to 9% and 11%, respectively, as compared to 5% in control cells. The increased ROS formation was associated with a concentration-dependent induction of HO-1 and GSTA mRNA (Figure 5B) and protein (Figure 5C) expression levels.

### Effect of VCX on Cell Autophagy

**Figure 6:**
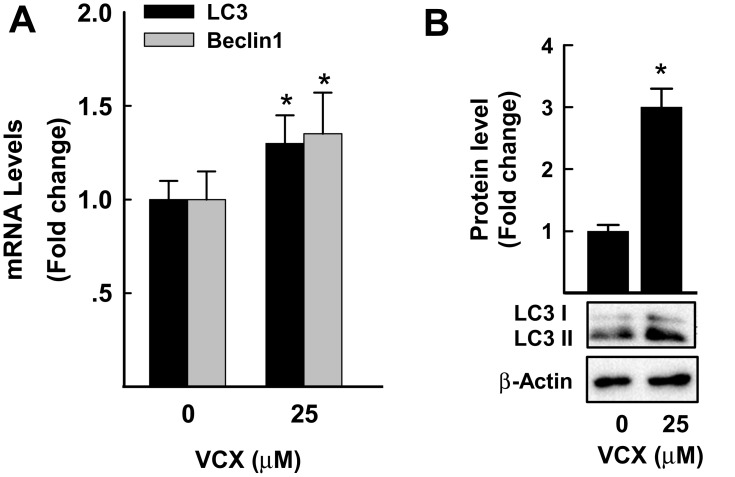
Effect of VCX treatment on autophagy in MDA-MB-231cells. MDA-MB-231 cells were treated for 24 h with VCX (25 μM). (**A**) LC3 and Beclin1 mRNA levels were quantified using qRT-PCR and normalized to β-actin housekeeping gene. The values are presented as mean ± SEM, (n = 6, triplicate). *P < 0.05 compared to control, VCX=0 μM, (Student’s *t*-test). (**B**) LC3I and II protein levels were determined by Western blot analysis. One of three representative experiments is shown. The values are presented as mean ± SEM. *p< 0.05 compared to control, VCX=0 μM, (Student’s *t*-test).

The possibility that VCX induces cell apoptosis via an autophagy-mediated cell death was tested by measuring the mRNA and protein expression of well-known markers of cell autophagy (LC3 and Beclin1)^27^ in response to VCX 25 μM. Figure 6A shows that VCX induced autophagy-associated cell death through increasing the mRNA expression of LC3 and beclin1 by approximately 30% and 40%, respectively. This was confirmed at the protein expression level, where VCX increased the expression of LC3II protein level by 3-fold (Figure 6B).

### Effect of VCX on the Chemo-Sensitivity of MDA-MB-231 Cells to DOX

**Figure 7:**
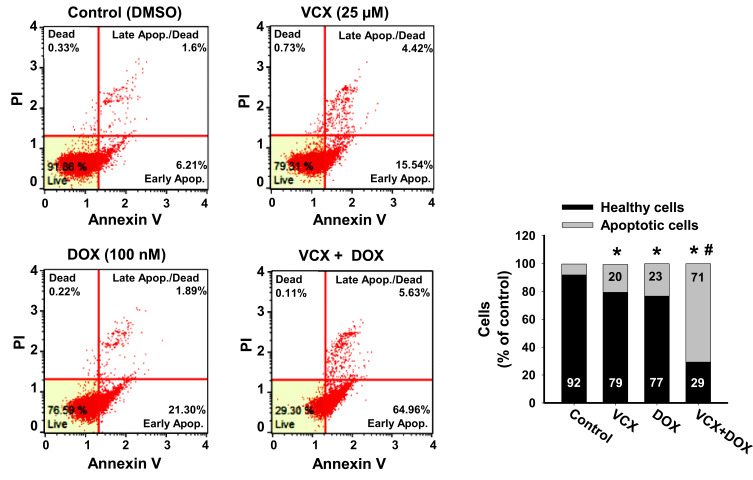
Effect of VCX on the chemo-sensitivity of MDA-MB-231 cells to DOX. MDA-MB-231 cells were treated for 24 h with VCX 25 μM alone or in combination with DOX 100 nM. Thereafter, the percentage of cell underwent apoptosis were determined using Muse^®^ Annexin V & Dead Cell Kit. The values are presented as mean, (n = 3, triplicate). *P < 0.05 compared to control, VCX=0 μM, ^#^*P* < 0.05 compared to DOX, (ANOVA followed by SNK test).

To test whether VCX would increase the chemo-sensitivity of MDA-MB-231 cells to chemotherapy, we examined the effect of VCX treatment in combination with DOX on MDA-MB-231 apoptosis level as a marker for chemo-sensitivity. Cells were treated for 24 h with VCX (25 μM) in the presence and absence of DOX (100 nM). Then, the percentage of cells that underwent apoptosis/necrosis was determined by flow cytometry. VCX or DOX monotherapy increased the percentage of apoptotic cells to 23% and 20%, respectively, as compared to control (8%). Importantly, DOX and VCX combination therapy synergistically increased the percentage of apoptotic cells to 71% as compared to DOX alone (Figure 7).

## Discussion

The predominant role of BCL-2 family is to regulate cell survival by modulating the integrity and the release of apoptogenic factors.^28^ Not surprisingly, BCL-2 overexpression has been linked to tumorigenesis initiation and the development of resistance to chemotherapies.^29^ Due to their regulatory roles in apoptosis, members of the BCL-2 family are considered as promising targets for cancer therapy.^28^,^29^ VCX is a potent and selective BCL-2 inhibitor with anti-tumor properties that usually correlates with its inhibitory effect on BCL-2. With increased BCL-2 expression in BC and particularly in TNBC, the possibility that VCX may suppress TNBC proliferation could not be ruled out. The available studies on the effect of VCX on BC cells proliferations only investigated the role of apoptosis, whereas the involvement of other mechanisms such as autophagy, cell cycle arrest, and oxidative stress has not been investigated. Therefore, the main objective of the current study was to explore the mechanistic role of autophagy, cell cycle arrest, and oxidative stress in VCX-induced breast cancer cell proliferation and growth inhibition.

The present study demonstrates the ability of VCX to cause a concentration-dependent cell growth inhibition in three breast cancer cell lines that pose different molecular subtypes, MCF-7 (ER+), SKBR-3 (HER-2+), and MDA-MB-231 (TNBC). Cell growth inhibition of VCX was more effective on both MCF-7 and SKBR-3 cells than on MDA-MB-231, with IC_50_ of approximately 35 μM for MCF-7 and SKBR-3 and 60 μM for MDA-MB-231 cells. The differential sensitivity of the cells to VCX could be attributed to several factors including the expression level of BCL-2. For example, BCL-2 is overexpressed in 85% of cases of luminal cancers (ER+),^30^ whereas overexpressed in approximately 41% of TNBC cases,^5^ which could explain the least sensitivity of MDA-MB-231 cells to VCX. The in vitro concentrations of VCX used in this study were maintained close to the therapeutic range of plasma concentration reported in humans.^31^ However, using higher VCX concentrations in the current study is attributed to the excessive concentrations of glucose, FBS, growth factors which all stimulate cell growth to promote breast cancer aggression and aggressiveness of TNBC.^32^ Other possible factors include the presence of an oncogenic mutation in the cell culture system, which reduces the efficacy of VCX concentrations required to achieve the anti-cancer effects.

In MDA-MB-231 cells, the ability of VCX treatment to induce mitochondrial dysfunction and membrane permeabilization is substantial evidence supporting VCX-induced apoptosis in a mitochondria-mediated pathway. The possibility that VCX exhibits anti-proliferative effects on MDA-MB-231 cells through induction of apoptosis was supported by the ability of VCX to a) increase the percentage of MDA-MB-231 cells undergo apoptosis and induce caspase 3/7 activity and gene expression at the mRNA and protein levels and b) induce the expression of several pro-apoptotic markers, caspase 3, caspase 7, BAX, while inhibit the expression of the anti-apoptotic BCL-2 with increased BAX/BCL-2 ratio. Our findings are in an agreement with previous observations, which showed that VCX induces apoptosis^10^ in breast cancer cells through BAX-dependent mechanism.^33^,^34^ Increased cell apoptosis by VCX in the current study was associated with induction of cell cycle arrest in G0/G1, leading to an increase in the percentage of cells in the G0/G1 phase with a decrease in the S phase cell population. The probable mechanism mediating the cell cycle arrest by VCX is the downregulation of positive regulators of G1-to-S transition including cyclin D1 though the inhibition of E2F1 transcription factor that is known to regulate cyclin D1 promotor.^35^ Although the exact mechanism of downregulation of cyclin D1 by VCX was not explored in this study, increased cyclin D1 degradation post-translationally by ubiquitination or phosphorylation is postulated,^36^ however further studies are needed. These findings agree with earlier studies showing that overexpression of cyclin D1 gene was observed steadily in breast tumors.^37^,^38^ In addition, it has been proposed that cellular senescence, which is a permanent cell cycle arrest in G0/G1 in response to different stressors, contributes to tumor supression.^39^,^40^

Another possible mechanism that could regulate VCX-induced apoptosis is oxidative stress.^41^ In this context, we report here that VCX-induced apoptosis was proportionally associated with a significant increase in ROS production and the expression of oxidative stress marker genes, HO-1 and GSTA, at both the mRNA and protein levels. Despite the fact that BCL-2 overexpression promotes cancer cell survival, recent reports provide evidence that the BCL-2 family also involves a redox component.^42^ Previous observations support our current results in which these studies showed that inhibition of cancer cells proliferation and induction of apoptosis and cell cycle arrest are mediated by induction of oxidative stress genes probably via the activation of ubiquitin-dependent proteasome degradation of cyclin D1.^43^ Importantly, induction of oxidative stress by VCX is believed to mediate the activation of autophagy-associated cell death pathway markers, LC3II and Beclin1, at the mRNA and protein levels. To our knowledge, this is the first report to demonstrate the inter-relationship between induction of apoptosis and autophagy-associated cell death by VCX-mediated inhibition of BCL-2 in MDA-MB-231 cells. In this regard, it was reported that Beclin1, an essential initiator of autophagy pathway, has a BCL-2 homology (BH3) domain. Interaction of BCL-2 protein with Beclin1 BH3 domain prevents assembling of the pre-autophagosome structure leading to autophagy inhibition, indicating that downregulation of BCL-2 by VCX is the first step toward activation of autophagy-associated cell death pathway.^44^ The dual induction of apoptosis and autophagic-associated cell death makes VCX an ideal chemotherapeutic target for breast cancer.

DOX and other anthracyclines trigger a DNA damage response and subsequently activate an apoptotic pathway to kill cancer cells.^45^,^46^ Since BCL-2 family plays a significant role in regulating apoptotic cell death, it is possible that inhibition of BCL-2 restores sensitivity to chemotherapeutic agents. The present findings show that the combination of VCX with DOX increases the chemo-sensitivity of MDA-MB-231 to DOX, and this could provide a new strategy to overcome the protective effects of BCL-2 and hence deliver efficacious therapies. These findings also suggest the potential of VCX to be used clinically to increase the efficacy of DOX and other chemotherapeutic agents.

## Conclusions

The present study demonstrates strong evidence that VCX a) induces human triple-negative breast cancer MDA-MB-231 cell growth inhibition through apoptosis, cell cycle arrest, autophagy-associated cell death, and oxidative stress mechanisms and b) increases the chemosensitivity to DOX in MDA-MB-231 cells.
